# Differentiated brand building of traditional mountain scenic spots in Jiangxi Province: Insights from tourists’ perception images and sentiment characteristics

**DOI:** 10.1371/journal.pone.0332121

**Published:** 2025-09-17

**Authors:** Yaguang Hu, Lei Yang, Dongbo Xu

**Affiliations:** 1 Jiangxi Modern Service Industry Development Research Institute, Jiangxi Flight University, Nanchang, China; 2 School of History, Nanjing University, Nanjing, China; 3 Center of National Security and National Defense Education, Nanjing Agricultural University, Nanjing, China; Zhejiang Agriculture and Forestry University: Zhejiang A and F University, CHINA

## Abstract

Understanding the perceptions and sentiment responses of tourists to traditional mountain scenic spots is crucial. This understanding is a cornerstone for improving brand image, optimizing space quality, and fostering sentiment connections between tourists and natural landscapes. Our study aims to address the homogenization issue in mountain scenic spots’ brand building. Using online comments and travel blogs as primary data, a mixed-methods approach integrating grounded theory, correspondence analysis, TF-IDF, sentiment analysis, and IPA analysis was employed. The result revealed several significant findings: (1) The tourist perception image represents a multidimensional dynamic system that includes resources, service, sightseeing, and time-space images. Different mountain scenic spots have contrasting characteristics regarding tourism perception images, with the sightseeing image proving a pivotal role in shaping these differences. (2) Among all mountain scenic spots, positive sentiment generally outweighs negative or neutral sentiments, while there are differences in sentiment preferences among perception images. (3) Interactions between tourists’ perception images and their sentiment are fundamental to the establishment of the brand of a scenic site. This research constructs a theoretical model for the generation path of tourism brand image, deepens the understanding of cognitive-affective interactions, improves the perception image system, and provides methodological support for big data-based tourism research. Hence, this study offers insights for the differentiated brand building and sustainable development of traditional mountain scenic spots.

## 1. Introduction

During the mass tourism era, governments at all levels in China are actively working to exploit regional tourism resources and cultivate tourism brands. Their efforts include improving cultural significance in tourism [1], developing seasonal tourism brands [2], and creating quality projects for regional tourist brands [3]. For example, some famous mountain scenic spots (MSSs) have incorporated Chinese revolutionary history and cultural history in brand creation procession, effectively creating diverse, rich perception images (PIs) and visitor sentiment experiences. This process cultivates unique cognitive memories and sentimental values among tourists. Finally, such strategies grant new competitive advantages to tourist destination brands.

Usually, natural scenic spots generate permanent online buzz or destinations that already possess world renown. However, tourism managers of these spots should focus on complex and intertwined issues, such as tourism experience, scenic area construction, and the ecological sustainability. Especially exploring hot issues and tourism marketing through natural environment seems to be a challenging job. However, these complexities make branding difficulty. Jiangxi Province is a relevant example with a wealth of natural tourism resources. MSSs represent a significant portion of these resources, accounting for 70%. By 2024, Jiangxi had 14 national 5A-level tourist attractions, which is the highest level in China’s scenic spot rating standards. Although these well-known mountain scenic spots have gained widespread recognition as “superior natural landscapes”, this superficial consensus may mask the inherent differences in their brand perception [4]. Therefore, these MSSs may fail to create a unique image in tourists’ perceptions. Thus, how to break through this homogenized perception and shape unique brand images for different MSSs has become a key research focus.

Furthermore, although relevant studies have been on the brand image and perception of scenic spots [5], only a few empirical studies have explored how tourism comments affect tourists’ perception of the destination and behavior [6]. The existing research indicate several trends. While related theories have matured, research paradigms and themes have become more diverse; there appears to be an emphasis on “cognitive image” over “sentiment image.” Comparative studies across different destinations remain rare. Although sentiment analysis is significant in business and society because it impacts strategic decision-making [7], there is still a lack of specific research investigating the composition of brand perception image (PI) and fine-grained sentiment analysis in MSSs.

To fill this gap and innovate research methods to deeply explore the authentic perceptions of tourists, we chose four of the most well-known MSSs in Jiangxi Province as research cases. Using the grounded theory paradigm and other methods, we analyze the online comments and travel blogs posted by tourists on five major online social communities over the past ten years. Compared with previous studies, we not only focused on tourists’ PI but also paid attention to the differences in sentiment expression behind them and conducted a comparative study. Our research sought to explore the following questions in depth:

Question 1: How can tourists’ PIs of MSSs be defined or concretized? What defines the characteristics and underlying generation logic of tourists’ PIs for MSSs?
Question 2: Based on tourists’ PIs, what is their overall sentiment toward different MSSs? What are the differences in sentiment characteristics across different dimensions of PIs? What are the key factors of positive and negative sentiments within the same dimension of PIs?
Question 3: Is it possible to construct a brand image generation model based on tourists’ PI for MSSs, and detail the transformation path of “perception image—sentiment interaction—tourism brand image competitiveness” be revealed?

Our study explores the correlation mechanism between tourists’ PIs and sentiment characteristics. The goal is to reveal explore the differentiated brand building path of traditional mountain scenic spots (MSSs) from the perspective of tourists’ perception images and sentiment characteristics, proposing actionable suggestions for enhancing the tourism brand image (TBI) of MSSs.

## 2. Literature review

### 2.1. Tourism brand image

TBI signifies the comprehensive impression and general feeling that tourist destinations impart to tourists [8]. It functions as a psychological picture formed by a set of characteristics defining destinations in various dimensions [9]. TBI reflects the perceptions, images, and sentiment-based assessments of tourists concerning the destination’s brand [10]. Therefore, TBI exert a critical impact on the success of a destination’s marketing efforts. It represents a significant avenue for destinations to compete effectively in the market and ensure a sustainable development of their scenic value.

Early research efforts have focused on evaluating the methods for constructing TBI. Strategies explored included shaping the image around urban historical and cultural backgrounds [11], unique cultural atmospheres [12], and world-class events organized in tourist destinations [13].

Later studies aimed to amplify TBI’s role in cultivating sustainable tourism development [14]. Meanwhile, the increasing influence of the internet prompted researchers to concentrate on the effect of marketing methods [15,16]. Correspondingly, researchers considered how destinations could differentiate themselves from similar scenic areas to achieve high attention and popularity [17,18]. Case studies verified the feasibility of these approaches, emphasizing such differentiation aimed at offering tourist unique sentiment-based experiences, thus strengthening their loyalty and sentiment attachment [19,20]. A large number of research has since accumulated, focusing specifically on the relationship between tourists and TBI. However, these studies paid little attention to MSSs, which demand distinct branding logics different from those of other tourism destinations.

In summary, the progress of TBI research is mainly based on theories related to influencing factors, space, and psychological patterns. Many research outcomes followed the first two theoretical domains, but the incorporation of sentiment interaction mechanisms into MSSs has not yet been thoroughly investigated.

### 2.2. Perception image of tourism destination

Although the image of tourism destinations plays a crucial role in shaping destination identity, this phenomenon remains understudied [21]. PIs reflect an individual’s cognition, perception, and impression of tourist destinations [22]; thus, shaping PIs is a key priority for tourism managers. PI significantly impacts tourists’ behaviors before, during, and after their travels [23,24], and is also related to their willingness to revisit and recommend the destination [25,26].

Recent studies have explored the influence of national strength, international status, and political stability on tourists’ PIs and intentions [27,28]. They also compared developed and developing countries and confirmed this correlation [29–31]. However, such macro perspectives cannot capture the MSSs’ PIs characteristics. Although studies consider PIs the most important part of brand equity theory [32], they can guide brand development [33].

With the development of new technologies, people have become accustomed to using search engines, social media, and APPs for communication [34], and online platforms have become the primary sources for tourists to obtain information [35]. Moreover, although social media and smart marketing are recognized as practical tools to enhance tourists’ PIs [36–39], existing studies lack a detailed analysis of how tourists’ online comments reflect different sentiment expressions across MSSs.

### 2.3. Tourist research

Tourists are the main participants in the tourism industry, and they can drive significant transformations and achieve sustainable development in tourism [40]. Studies related to tourists mainly focus on three topics.

The first topic is tourists’ consumption behavior. Specifically, self-congruity theory explores the alignment between tourists and destinations [41]. Studies have found that self-consistency is closely related to the social environment and culture [42], and can strongly impact personal behaviors, including tourists’ purchase intentions and motivations [43]. Furthermore, when the symbolic meaning of a tourism product is perceived to be consistent with the self-congruity of the target consumers [44], it motivates consumption behavior and can be regarded as a precursor to consumption [45]. Additionally, self-congruity impacts consumer satisfaction and tourists’ perception of brand equity [46]. However, few studies have investigated how this consistency is shaped by MSSs’ unique brand perceptions, which limits the understanding of how MSS features drive tourist behaviors.

The second topic is tourist loyalty. Literature indicates that repeat customers support the sustainable development of tourism, with personal loyalty to a destination being a key factor [47]. Thus, loyalty is generally regarded as a key indicator for a successful destination [48], and it is also a significant marketing strategy for the tourism industry [49]. Moreover, tourist loyalty stems from positive recommendations from others [50]. Therefore, in the context of well-developed online marketing, it is necessary to pay close attention to and reasonably analyze the tourism reviews posted by tourists online.

The third topic is tourists’ sentiment. The research is generally integrative, based on studies of tourists’ consumption behavior, psychology, or human-environment interaction, using questionnaire surveys, platform data, or travel experiences to explore the relationship between tourists’ sentiment and tourism development [51]. Studies show that positive sentiment can drive tourists’ pro-environmental behaviors, even outweighing factors such as rationality and morality [52]. Meanwhile, situations such as overcrowding can trigger negative sentiment [53], leading tourists to develop avoidance intentions toward the destination, while negative online sentiment exerts an even broader impact [54]. Studies point out that high-quality interactions can foster a sense of unity [55], and stress-free services as well as opportunities for tourists to express themselves can promote positive sentiment interactions and enhance satisfaction [56]. It is essential to note that not all scenic spots should be treated equally [57]. However, existing studies rarely compare sentiment interactions between famous MSSs with similar reputations, which makes it difficult to determine the differentiation codes’ critical for their sustainable competitiveness. This is the gap our comparative study aims to address.

## 3. Research design

### 3.1. Overview of research case

To assist MSSs in making a breakthrough in brand construction, our study, taking into account the location distribution, scenic spot’s level, passenger flow, honors and scenic characteristics of famous MSSs in China, we decided to take Jinggang Mountain, Longhu Mountain, Lushan Mountain, and Sanqing Mountain as the research cases. Below are three significant research advantages:

These cases typically exhibit the following characteristics. As a major concentration of MSSs in eastern China, it encompasses revolutionary historical mountains, Danxia landform spectacles, cultural mountains, and ecological environment mountains, covering the main types of MSSs in China. These are highly consistent with nationwide MSS resources, making the research results highly representative.

These cases face common challenges in brand building. Although the types of MSSs in Jiangxi Province are diverse, they may still be troubled by homogeneous competition, such as overlapping natural landscapes. This predicament is highly consistent with the current situation of other MSSs in China, especially in regions with similar resource concentrations. Therefore, this research has universal reference value.

The feasibility and completeness of the data. These cases are all 5A-level scenic spots, the highest level in China’s rating system, with annual passenger flow ranking among China’s top ten famous mountains. The comments and travel blogs tourists post on online platforms are relatively abundant, providing sufficient samples for the research and ensuring the reliability of the findings.

### 3.2. Data collection and processing

Online comments and travel blogs from tourists are the main data sources for our study. Data was collected from five major tourism-related social platforms in China: C-trip (https://www.ctrip.com/), Qunar (https://www.qunar.com/), DianPing (https://www.dianping.com/), Mafengwo (https://www.mafengwo.cn/), and Tongcheng Travel (https://www.ly.com/). We selected these platforms for two key reasons:

**Broad user and high-quality comments:** The remaining platforms are highly popular website for travel guides and entertainment, integrate tourism services, travel blogs, and consumer reviews, and they all feature user and active user rankings among the top travel websites in China. Therefore, these platforms have a high reference value. In addition, they encourage users to publish detailed and in-depth comments in various formats, including text, images and videos. This facilitates the collection of comprehensive tourist feedback and a better understanding of the true feelings of tourists.

**Compliant data collection and processing:** No specific permits were required for getting all the online comments data, as all the data from these online platforms did not involve human participants, human specimens or tissue, vertebrate animals or cephalopods, vertebrate embryos or tissues or fields research. Further, there is no conflict of interest in the choice of data source, as all data comes from a public website and is freely available to the public. Furthermore, our study did not obtain any non-public information or copyrighted proprietary content from the aforementioned online platforms.

After confirming the data source platform, we used the Octopus Collector to collect online reviews related to four MSSs. The Octopus Collector is a commonly used data collection tool in China [58], compatible with most Chinese websites, supporting Chinese, and capable of collecting within a specific period. Therefore, it meets our research requirements.

The data collection and analysis methods used in our study adhered to the terms and conditions of the data source to ensure compliance and ethical standards.

When setting up the Octopus Collector, the time range was set from January 2015 to September 2024 to ensure coverage of a long period, including different seasons and holidays. For data output, we conducted data cleaning based on the following four criteria: (1) deleting the same content submitted from the same IP address; (2) deleting meaningless content with fewer than five characters; (3) eliminating identical copy-pasted texts; (4) excluding advertisements, poems, and invalid data. After data cleaning, the valid comments obtained for four MSSs are shown in Table 1.

**Table 1:** Overview of valid data of four MSSs.

	Jinggang mountain	Longhu mountain	Lushan mountain	Sanqing mountain	Total
Number of comments	2095	3422	6523	3313	15353
Number of travel blogs	67	63	113	82	325

**Fig 1:**
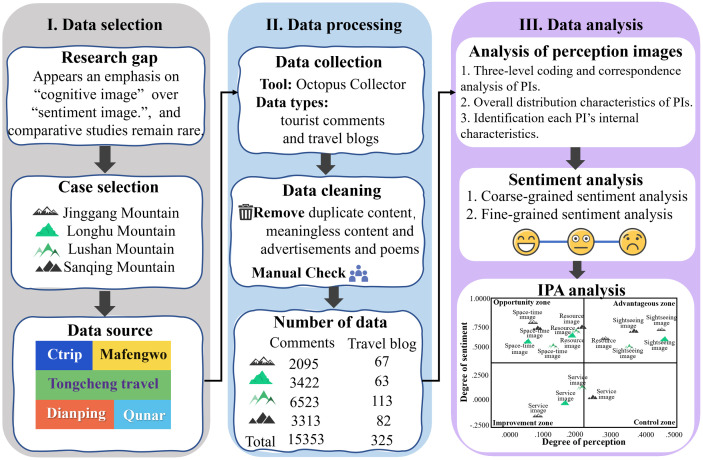
The research route of this article.

Fig 1 presents the roadmap of our research, which adopts a comprehensive research methodology and focuses on qualitative analysis, aiming to address three components: a holistic analysis of tourists’ PIs, sentiment analysis of tourists’ PIs, and an IPA analysis. These components are operationalized through three research steps. Step 1 addresses the first component, Step 2 corresponds to the second, and Step 3 tackles the third.

### 3.3. Grounded theory and correspondence analysis

Data relevant to the study were collected based on the principles of grounded theory, which can help to identify the main issues that participants focus on during their shared experiences [59]. Our research used ROST CM 6.0 software. It is a commonly used analytical tool for Chinese text and integrates functions for word segmentation, word frequency analysis, and statistics [60]. We developed a coding scheme based on grounded theory, utilizing a “bottom-up” deductive method to group high-frequency words, reduce dimensions, improve categories, and transcribe the data to reveal the complex dynamic framework of the PI. The coding team consists of 2 coders with a tourism management professional background and rich text coding experience. Additionally, the team adopted a mutual agreement degree method to measure the internal reliability of the coders, which meets the reliability requirements of the analysis [61], with the formula as follows:


$$K=\frac{\text{2}M}{\left(N_{\text{1}}+N_{\text{2}}\right)}$$ (1)

where K presents the mutual agreement, M denotes the number of coding agreements between coders, and *N1, N2* present the total number of coding by coders. Discussions were held to reach a consensus on inconsistent codes. Formal coding began only when the inter-coder reliability reached above 0.8.

The specific steps are: (1) Use the ROST CM 6.0 to identify the top 140 high-frequency words across all MSSs. (2) To reduce coding bias, the coders were asked to pre-code 50 high-frequency words randomly selected from MSSs’ comments and utilize the mutual agreement degree method. (3) The coders independently code the four PIs, i.e., resource image, sightseeing image, service image, and space-time image, and then classify them. The clustering process of conceptual categorization was applied to identify four core concepts (tertiary code). These core concepts further comprised 14 related (secondary code) and 85 initial (primary code) concepts. Coders shall discuss any differences of opinion together to reach a decision. Other professors will be invited to join the deliberation if a consensus cannot be reached after the discussion. (4) Compare whether there are differences in PIs among the four MSSs and within each MSS.

### 3.4. Sentiment analysis

The research applied coarse-grained and fine-grained sentiment analysis on PIs. We used SPSSAU for the coarse-grained sentiment analysis, a widely used web-based data statistics tool [62] that includes pre-built sentiment lexicons for semantic matching [63]. We calculated sentiment scores for each comment, ranging from −1 to 1.

Furthermore, we employed fine-grained sentiment analysis to capture the dynamic changes in sentiment through tourists’ comments. Specifically, Wenxin 4.0 Turbo (a large language model, LLM) leverages robust natural language processing functions and a pre-trained model to categorize 15,353 comments and identify their core PI types. Although LLM is generally regarded as an effective tool for handling natural language processing [64], the team still adopted a mutual agreement degree method to ensure the reliability of the classification results. An independent researcher randomly selected 400 comments for manual classification and then calculated the level of agreement with LLM’s results. A value exceeding 0.8 indicates good reliability. After completing these classifications and obtaining a corpus of comment texts classified into four PIs, we calculated the average sentiment score for each type of PI within each MSS, ultimately achieving fine-grained sentiment computation at the PI level.

The steps were:

(1) We used prompts and pre-trained Wenxin 4.0 Turbo to define the PIs. We optimized the prompts through multiple attempts, enabling the classification of the core perceived image categories for all comments. The final prompt is presented in Table 2.

**Table 2:** The prompt when utilizing the Wenxin 4.0 Turbo.

Sample of deep data categorization for comments
I am an information coder. The attachment contains tourists’ comments about Jinggang Mountain, where the first column is the project number and the second column is the comment content. According to the definitions of the following four perception images:
1. Resources image refers to tourists’ sensory impressions of the natural and humanistic resources of a tourist destination;
2. Sightseeing image refers to tourists’ embodied cognition and psychological experience during their tour in the scenic area;
3. Service image represents tourists’ feelings and evaluations of various service systems in the tourist attraction, covering the service levels of the tourist destination in multiple aspects such as ‘food, accommodation, transportation, travel, shopping, and entertainment’;
4. Space-time image represents the sum of tourists’ comprehensive perceptions and cognitions of the geographical spatial environment of MSSs and various tourism activities and scenes under different time dimensions during their tour.
Please classify the 2095 pieces of information in the attachment one by one. No explanation is needed, and only the corresponding image type name needs to be generated after each project number.

After inputting the definition prompts for the four PIs into Wenxin, it uses the text classification function to categorize all tourist comments into the predefined categories, and outputs the text datasets corresponding to the four PIs for each sample, respectively.

(2) Utilized the sentiment analysis function module of SPSSAU to conduct sentiment analysis on a row basis. Using a sentiment dictionary, the sentiment scores of the comment text data for the four PIs were calculated sequentially, with the sentiment scores compressed between −1 and 1.
(3) We combined the Term Frequency-Inverse Document Frequency (TF-IDF) algorithm, a widely used weighting technique in information retrieval and text mining. It aims to extract positive and negative sentiment feature words related to different perceived images across various MSSs, thereby presenting the granularity and dynamic shifts in tourists’ positive and negative sentiments. The formulas are as follows:

Term Frequency (TF) indicates the number of times a word appears in a specific document.


$${TF}_{ij}=\frac{\text{Number of occurrences of the term }i\text{ in document }j}{\text{Total number of terms in document }j}$$ (2)

Inverse Document Frequency (IDF) indicates the universality of a word in the entire corpus.


$${IDF}_{i}=\text{log }\frac{\text{Total number of documents}}{\text{Number of documents containing term }i+\text{1}}$$ (3)

Selecting words with the highest TF-IDF values as the topics of a document can significantly improve accuracy.


$$\text{TF}-{\text{IDF}}_{ij}={\text{TF}}_{ij}\times {\text{IDF}}_{i}$$ (4)

### 3.5. IPA analysis

To better understand tourists’ value judgments and image positioning related to their PIs for each MSS, we used Importance-performance analysis (IPA) for reference, one of the most widely used tools in tourism research. We typically utilized to identify discrepancies between stakeholders’ perceptions of the importance of specific components within a given issue and their evaluations of the actual management performance concerning those components [65]. This method generally constructs a two-dimensional coordinate graph where “importance” is on the horizontal axis and “actual performance” is on the vertical axis. It divides the data points into four quadrants, visually presenting each attribute’s performance characteristics.

The steps were: (1) Replace the indicators and determine the benchmark point. Replace “importance” (I) and “actual performance” (P) in the original model with “degree of sentiment” and “degree of perception”, respectively. Calculate the average values of all observed variables based on the sentiment degree and intensity, and use these data to determine the benchmark intersection point in the IPA figure. According to this point, draw a cross coordinate axis where the horizontal axis represents the degree of perception and the vertical axis represents the degree of sentiment. (2) Positioning of observed variables: Map the four MSSs’ core PIs degree and sentiment degree into the four quadrants to explore the actual status of tourists’ PIs of TBI.

## 4. Results

### 4.1. Analysis of PIs on MSSs

#### 4.1.1. Identify three level code of PI and corresponding analysis.

**Fig 2:**
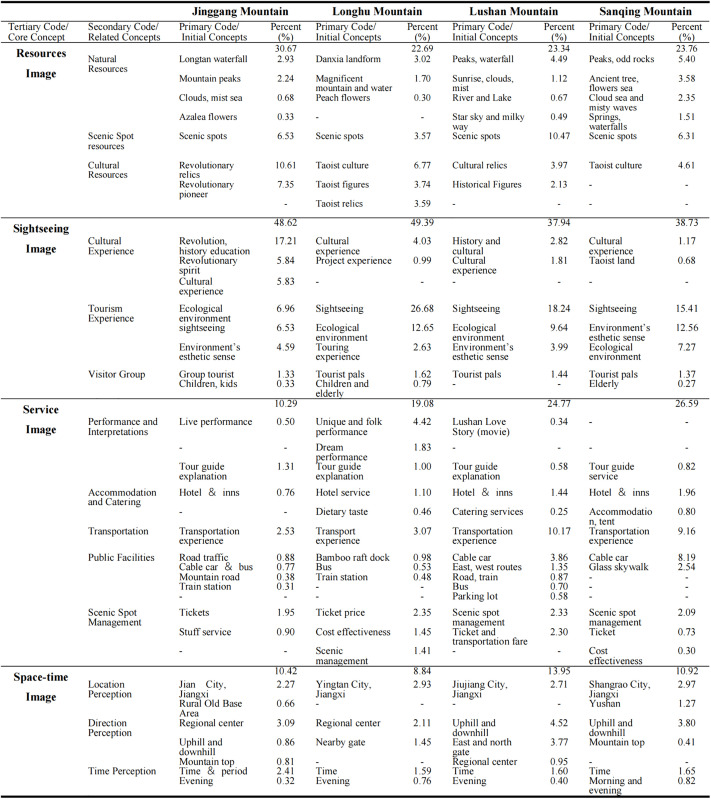
Distribution of PIs encoding ratios of MSSs.

Based on grounded theory, we used ROST CM 6.0 to code the high-frequency words in the comments, identified four PIs of tourists towards MSSs, and calculated the word frequency percentage of different concepts to reflect the degree of tourists’ attention to each perception image category. Moreover, we calculated the percentage values of word frequency for different concepts. The higher percentage value suggests greater tourist attention towards that particular category. The relevant statistical results laid the data foundation for subsequent analyses. The statistical results are shown in Fig 2.

Resources image refers to tourists’ sense impression of natural and cultural resources in tourist destination. Sightseeing image refers to the self-cognition and psychological experience of tourists in sightseeing process. Service image represents the tourists’ evaluation of the service system of the tourism scenic spot, enabling the service level of the destination in many aspects such as accommodation, travel, shopping and entertainment. The space-time image represents tourists’ comprehensive perceptions and cognitions of MSSs regarding full time (daytime, nighttime, and other periods), the entire region (uphill, downhill, and other areas), and full seasons.

**Fig 3:**
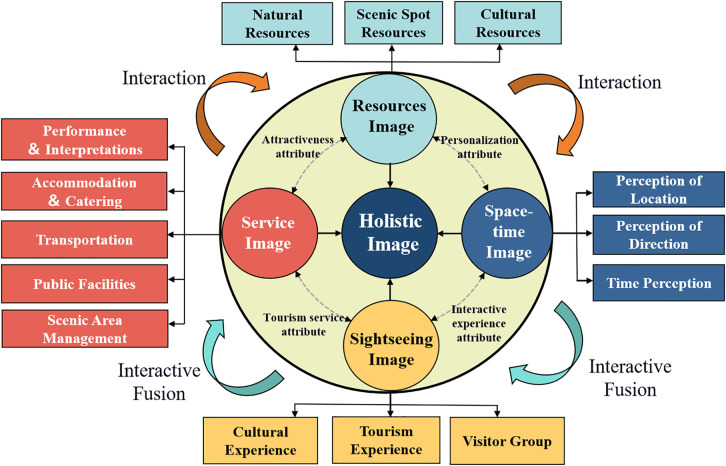
Conceptual model of PIs.

Drawing upon the core and related concepts detailed in Fig 2, a conceptual model of PIs has been developed, as illustrated in Fig 3. Upon comparison, the model testifies to a comprehensive coverage of all data information. This indicates that the conceptual model has reached saturation.

In addition, a cross-tabulation table is constructed utilizing frequencies of four core concepts and four MSSs as the X and Y axes. To examine the correspondence between PIs and MSSs, correspondence analysis was employed. The chi-square test yielded a Pearson’s chi-square value of 3141.595 (p < 0.001), indicating a significant correlation between the four MSSs and PIs, thus verifying the analysis. Besides, through dimensionality reduction, the singular value of Dimension 1 was determined to be 0.144 (inertia = 0.021), explaining 84.90% of the variance in cross-tabulation table. The singular value of Dimension 2 is 0.060 (inertia = 0.004), accounting for 14.8% of the variance. These two dimensions accounted for 99.7% of the information in the cross-tabulation table, indicating their effectiveness in representing the perception relationship between PIs and MSSs.

**Fig 4:**
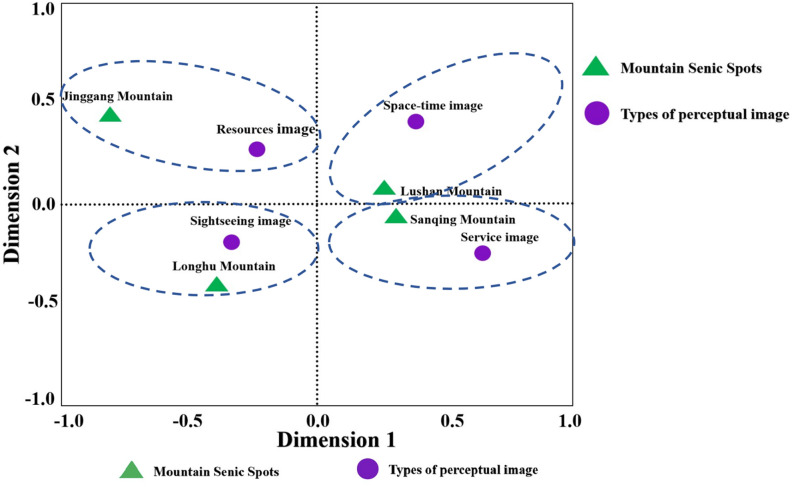
Distribution diagram of the correspondence.

Fig 4 illustrates the distribution of PIs and MSSs across four quadrants, showing significant differences in PIs among the four MSSs. This distribution further confirms that, from the perspective of tourist PIs, the image characteristics of MSSs can be effectively distinguished.

#### 4.1.2. Overall distribution of PIs.

Preceding research has addressed the three-level code of PIs. However, a question remains: could there be discrepancies in PIs even among scenic spots of the same type? To investigate this, we developed Table 3, which ranks the proportion of PIs codes.

**Table 3:** PIs Coding Proportion of MSSs.

	Resources Image	Ranking	Sightseeing Image	Ranking	Service Image	Ranking	Space-time Image	Ranking
Jinggang mountain	30.67	1	48.62	2	10.29	4	10.42	3
Longhu mountain	22.69	4	49.39	1	19.08	3	8.84	4
Lushan mountain	23.34	3	37.94	4	24.77	2	13.95	1
Sanqing mountain	23.76	2	38.73	3	26.59	1	10.92	2
Mean	25.12	2	43.67	1	20.18	3	11.03	4

Note: Figures are in percentages.

Table 3 indicated the following characteristics in the distribution of PIs among four MSSs:

Firstly, the sightseeing image category dominates in PIs, with a mean value of 43.67%. This dominance suggests that during the construction of PIs, it is important to emphasize the participation of tourists in local culture and nature. By progressing through phases of “participatory perception, procedural cognition, and motion consciousness,” the brand image of tourist destinations can be deepened.

Secondly, compared to sightseeing image, the proportions of the remaining three categories are notably lower. This observation implies that despite the years of development of these MSSs, considerable potential for improvement remains, particularly in relation to resources, service, and space-time image.

Despite being categorized as MSSs, these destinations exhibit inconsistent performance across specific image dimensions. Jinggang mountain dominates in resources image, whereas Longhu mountain excels in sightseeing image. Sanqing mountain demonstrates the strongest performance in service image, and Lushan mountain’s space-time image leaves the most lasting impression on tourists. Therefore, this research offers a perspective for optimizing tourist PI strategies tailored to each MSSs.

#### 4.1.3. Dimensional Identification of PIs.

The above study analyzed the percentages of the four core concepts (Tertiary Code). To better understand the characteristic of three level code, we conducted a detailed analysis of the internal features of these four core concepts and made a horizontal comparison of the proportions of related concepts (secondary codes) to illustrate the differences in the internal composition of different core concepts.

**Fig 5:**
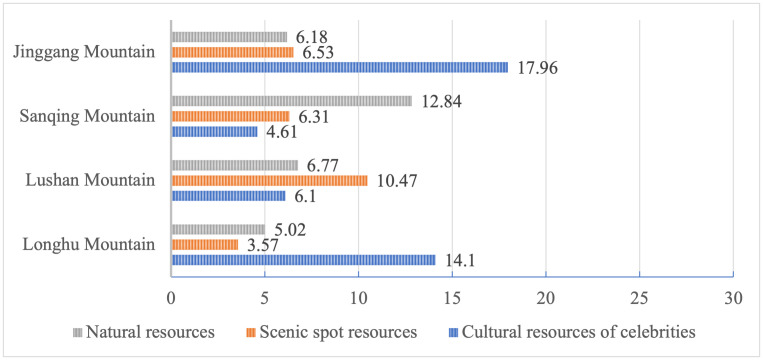
Distribution of encoding in resources image. Note: Figures are in percentages.

Fig 5 offers a comparative analysis of the secondary code “resources image,” indicating that Sanqing Mountain has the most significant natural resource advantages. Lushan mountain is notable for tourists’ increased perception of its scenic spot resources, evidenced by frequent citations of scenic spot names in travel blogs. Due to its historical and revolutionary relics, Jinggang mountain exhibits the highest proportion of cultural resources. Specifically, while both Sanqing mountain and Longhu mountain emphasize Taoist culture, Longhu mountain demonstrates a greater proportion, suggesting it usually conveys richer Taoist cultural experiences.

**Fig 6:**
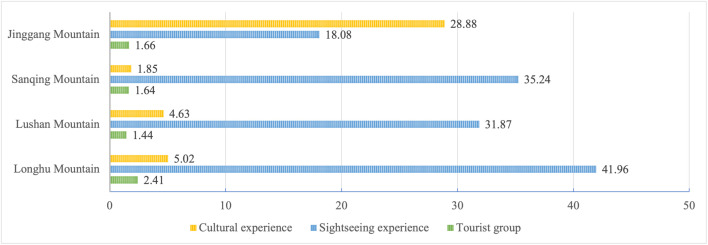
Distribution of encoding in sightseeing image. Note: Figures are in percentages.

Fig 6 reports the “sightseeing image.” In this category, Jinggang mountain demonstrates the most significant cultural experience, with comments frequently featuring keywords such as “significant effect,” “spiritual purification,” “Jinggang mountain spirit,” and “revolutionary history,” aligning with its TBI. Longhu mountain exhibits the greatest advantages in tourism experiences, as comments commonly include phrases such as “picture-like scenery,” “comfortable,” and “relaxing.”, as well as strong recommendations for “bamboo rafting on Luxi River,” indicating that this activity creates a unique tourism image for visitors. Furthermore, regarding tourist demographics, while terms such as “tourists,” “children,” and “pals” are mentioned in all MSSs, Jinggang mountain demonstrates a higher frequency of references to “group tourist” and “colleagues.” In comparison, Sanqing mountain and Longhu mountain repeatedly mention “elderly,” suggesting demographic differences remain even among similar types of MSSs.

**Fig 7:**
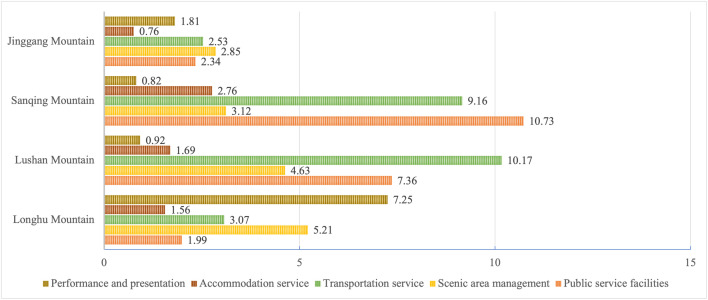
Distribution of encoding in service image. Note: Figures are in percentages.

Fig 7 offers an analysis of “service image.” Longhu mountain exhibits a significantly greater advantage in performance and interpretation compared to the other MSSs, primarily attributed to its folk cultural live demonstrates, while Sanqing mountain shows a less significant effects in this code but excels in accommodation and carting services, with keywords such as “homestay,” “tent camping,” and “Hilton” frequently appearing in comments. Lushan and Sanqing mountains are particularly strong in transportation and public facilities, largely due to their unique services such as “cable car,” “glass skywalk,” and “bus.” Longhu mountain features unique water transportation options, often referring to “bamboo raft,” “dock,” and “boatman.” Regarding scenic spot management, all destinations perform relatively uniformly, as tourists appear sensitive to internal management, staff services, ticket and transportation fares, and queuing procedures.

**Fig 8:**
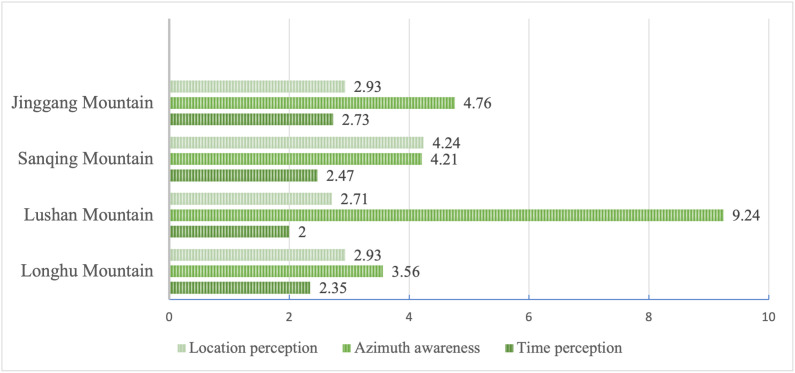
Distribution of encoding in space-time image. Note: Figures are in percentages.

Fig 8 offers an analysis of “space-time image.” Sanqing mountain demonstrates the highest ranking in location perception, attributed to its unique geographical positioning at the intersection of three provinces, and at the intersection of several economic and ecological areas in China. Lushan mountain has a prominent direction perception, with comments frequently including keywords such as “uphill and downhill,” “East Gate,” and “North Gate.” These are attributed to Lushan Mountain’s multi-space integrated tourism structure. For example, Lushan West Sea and Lushan Mountain, which are 80 km apart, have been highly integrated. This has greatly enhanced tourists’ perception of space. However, the current practice of characteristic planning for four MSSs in different seasons is still insufficient, leading to strong perceptions of special periods, such as holidays, but is weak in different seasons.

### 4.2. Sentiment analysis of PIs

#### 4.2.1. Coarse-grained sentiment analysis of MSSs.

Tourist destination sentiment is understood to be constructed upon cognitive orientation, drawing from the “cognitive-affective” image theory [66,67]. Favorable destination images can bring about positive PI or behavioral outcomes related to the destination [68]. Usually, the individuals’ sentiment impression of a destination is typically measured on semantic differential scales [69]. We used the sentiment analysis module in SPSSAU to conduct coarse-grained analysis to better process Chinese textual data.

Table 4 described tourists’ sentiment orientations and scores for each MSS. In general, the percentage of comments and sentiment scores across the four MSSs were quite similar, with positive comments more frequent than negative comments. Among these MSSs, Jinggang mountain displayed the highest proportion of positive comments.

**Table 4:** Comparison of PIs and sentiments in four MSSs.

	Positive			Negative			Neutral		
	NOC	Percent (%)	ASS	NOC	Percent (%)	ASS	NOC	Percent (%)	ASS
**Jinggang Mountain**	1685	80.43	0.92	260	12.41	−0.83	150	7.16	0.03
**Longhu Mountain**	2647	77.36	0.92	538	15.72	−0.85	237	6.92	0.03
**Lushan Mountain**	4687	71.86	0.89	1205	18.47	−0.81	631	9.67	0.01
**Sanqing Mountain**	2530	76.37	0.91	495	14.94	−0.83	288	8.69	0.04

Note: NOC means “Number of comments”. ASS means “Average Sentiment Score”.

#### 4.2.2. Fine-grained sentiment analysis of MSSs.

The criteria for judging the sentiment orientation are as follows: [−1, −1/3) represents negative sentiment, [−1/3, 0) represents slightly negative sentiment, [0, 1/3) represents slightly positive sentiment, [1/3, 1] represents positive sentiment. The average value of the sentiment degree for each PI in each MSS was calculated successively.

As presented in Table 5, tourists demonstrate heterogenous sentimental preferences for different MSSs across various PIs. Specifically, Sanqing mountain exhibits the strongest sentiment preference for the resources image, while Jinggang mountain demonstrates the most significant sentiment preference for both the sightseeing and space-time images; whereas, Lushan mountain demonstrates the strongest preference for the service image.

**Table 5:** The average sentiment scores for different MSSs across four core concepts.

	Resources Image	Ranking	Sightseeing Image	Ranking	Service Image	Ranking	Space-time Image	Ranking
Jinggang mountain	0.63	2	0.69	1	−0.31	4	0.82	1
Longhu mountain	0.58	4	0.61	3	−0.14	3	0.61	3
Lushan mountain	0.59	3	0.47	4	0.10	1	0.55	4
Sanqing mountain	0.65	1	0.62	2	−0.01	2	0.77	2

Note: The range of sentiment mean values is from [−1, 1]. “-1” represent negative sentiment. “1” represent positive sentiment, with “0” indicating neutral sentiment.

It should be acknowledged that prior research has often overlooked the analysis of sentimental differences in the same core concept of PIs. Besides, high-frequency word statistics frequently emphasize static results, such as place names and attractions [70], thereby diluting the key vocabulary and sentiment variations perceived by tourists [71]. To address these limitations and gain deeper insights, we introduced TF-IDF analysis into our study. Compared to methods based solely on word frequency, TF-IDF analysis prioritizes keywords with higher semantic importance [72], aiming to identify the sentiment tendency of tourists [73]. Therefore, we proceeded to sequentially extract positive and negative sentiment keywords with relatively high TF-IDF weights from each MSS. Our aim was to clearly illustrate the characteristics of sentimental distribution specifically across each core concept of PIs.

Table 6 shows the results of TF-IDF analysis, we can draw main findings are as follows:

**Table 6:** The average sentiment scores for different MSSs across four core concepts.

		Positive Sentiment (TF-IDF Weight)	Negative Sentiment (TF-IDF Weight)
**Resources Image**	**Jinggang mountain**	Picture scene 0.48, great 0.47, memorial tower 0.41, pleasant climate 0.40, good landscape 0.39, fresh air 0.39, classic 0.39, first-class 0.39, beautiful 0.38, pleasant scenic 0.37	Hazy 0.35, nothing special 0.31, heavy fog 0.29, mist 0.28
	**Longhu mountain**	Picture scene 0.43, fresh air 0.40, beautiful view 0.37, clear waters and lush mountains 0.37, good reputation 0.36, green mountain and clear water 0.36, good view 0.34, breathtaking beauty 0.34, rich culture 0.31, beautiful landscape 0.30	Just so-so 0.32
	**Lushan mountain**	Picture scene 0.49, pleasant scenic 0.43, very beautiful 0.43, fresh air 0.39, good view 0.38, good landscape 0.37, elegant 0.37, beautiful scenery 0.36, fresh air 0.36, breathtaking beauty 0.34, good reputation 0.34	Little water for waterfall 0.36, heavy rain 0.30, foggy 0.29
	**Sanqing mountain**	Good environment 0.49, pleasant scenic 0.44, beautiful scenery 0.39, picture scene0.37, natural landscape 0.35, great scenic 0.34, like a picture0.34, very beautiful 0.34, clear waters and lush mountains 0.33, fairyland 0.33	Bad weather 0.39, rainy day 0.27, heavy rain 0.27, rain and mist 0.26, hazy 0.25
**Sightseeing Image**	**Jinggang mountain**	Deeply moved 0.48, passionate 0.46, pleasant 0.45, tour of study 0.38, solemn and respectful 0.36, leisure 0.36, fun 0.35, land of spirit 0.34, inheritance 0.33, vacation 0.32	Regret 0.50, tiring0.34
	**Longhu mountain**	Quite fun 0.54, nice 0.42, interesting 0.38, wonderful 0.37, very suitable 0.33, worth to trip 0.33, great location0.33, pleasant 0.32, satisfied 0.31, truly recommended 0.31	A bit tired 0.54, too hot 0.29, too tired 0.28, very tired 0.25
	**Lushan mountain**	Interesting 0.44, satisfied 0.42, have fun 0.41, relaxed and refreshed 0.41, fun 0.39, worth to trip 0.38, very suitable 0.35, meaningful 0.35, happy 0.34, worth the trip 0.34	Exhausted 0.40, too tired 0.38, very tired 0.29
	**Sanqing mountain**	Will come again 0.51, interesting 0.47, worth seeing 0.40, happy 0.39, nice 0.37, fun 0.37, local style 0.32, cool 0.30, pleasant 0.30, comfortable 0.30	A bit tired 0.37, quite tired 0.34, can’t climb anymore 0.33, fear of heights 0.26
**Service Image**	**Jinggang mountain**	Discount 0.46, ticket issue 0.39, staff attitude 0.38, hygiene 0.38, cost effective 0.32, convenient0.30, good work 0.30	Too expensive 0.39, rip-off 0.34, washroom 0.32, crowded 0.32, closed time 0.30, ticket issue 0.30, waste time 0.29, troublesome 0.27, commercialized 0.27, management 0.27
	**Longhu mountain**	Convenient and fast 0.45, room 0.31, good value 0.31, self-service 0.29, good work 0.29, tourist-friendly 0.27, performance 0.25	High price 0.45, ticket price 0.43, too expensive 0.35, scalper 0.35, stuff attitude 0.33, overcrowded0.32, waiting for bus 0.31, service awareness 0.28, ticket issue 0.27, commercialization 0.26
	**Lushan mountain**	Fast 0.57, okay 0.53, convenient and fast 0.49, ticket issuance 0.47, discount 0.39, cost effective 0.36, well-developed 0.35, convenient 0.340, reservation 0.34, ticket exchange 0.33	Maintenance 0.38, stuff attitude 0.37, hygiene 0.36, rip-off 0.36, overcrowded 0.34, unreasonable 0.34, need improvement 0.34, ticket price 0.33, mass management 0.30, too expensive0.30
	**Sanqing mountain**	Convenient and fast 0.63, okay 0.48, good job 0.46, smooth 0.44, convenient 0.41, satisfied 0.38, fast 0.37, enthusiastic 0.32, well-developed 0.31, cost-effective 0.31	Too expensive 0.38, cutting in line 0.33, need improvement 0.32, staff attitude 0.32, washroom 0.32, road sign 0.30, support facilities 0.29, overcrowded 0.29, parking fee 0.27, cable car capacity 0.26
**Space-time Image**	**Jinggang mountain**	Surroundings 0.30, season 0.29, evening 0.15	International Labor Day 0.37, holiday 0.33, too far 0.29, scattered 0.28
	**Longhu mountain**	Quite large 0.33, daytime 0.31, spring 0.30, season 0.29, Shangrao city 0.28	Holiday 0.26
	**Lushan mountain**	South of the mountain 0.35, autumn 0.31, summer 0.30, all-season 0.29, side of the ridge 0.29, evening 0.17	Rainy season 0.32, dry-water season 0.31, too far 0.30
	**Sanqing mountain**	Service area 0.26, season 0.22, downhill 0.13, uphill 0.13	Chinese New Year 0.28, peak season 0.17

(1) For the sentiment characteristics associated with the resources image, positive sentiments dominate overwhelmingly. This dominance is supported by the unique natural environment and rich cultural heritage, all contributing to positive sentiment responses. However, negative sentiments also occured, often related to weather conditions, such as instances of “heavy rain” and “foggy”.
(2) Regarding the sightseeing image, sentiments are generated through tourists’ participation and experiences, both cultural and touristic, due to their engagement with resources. Representative keywords indicating positive sentiments include terms such as “deeply moved,” “interesting,” and “will come again.” Compared to other words, there is also a smaller set of negative words, like “regret” and “tired”.
(3) The service image is commonly represented by negative sentiments. Negative evaluations are mainly based on aspects such as ticket prices, available facilities, commercial atmosphere, quality of staff service, and transport related issue.
(4) Concerning the space-time image, sentiment characteristics are relatively less significant. Positive sentiments tend to focus on the sense of space and the scenic qualities on different seasons. Negative sentiments, on the other hand, are mainly associated with holidays, possibly due to the overcrowding of tourists during these peak seasons and special stages.

These findings suggest that positive and negative sentiments are not uniform and can vary across different PIs. Moreover, even when considering a single dimension of PIs, the characteristics exhibit a spectrum of connection and divergence. This variability effectively illustrates the complexity, dynamism, and interconnectedness that define PIs.

### 4.3. IPA analysis of PIs in MSSs

**Fig 9:**
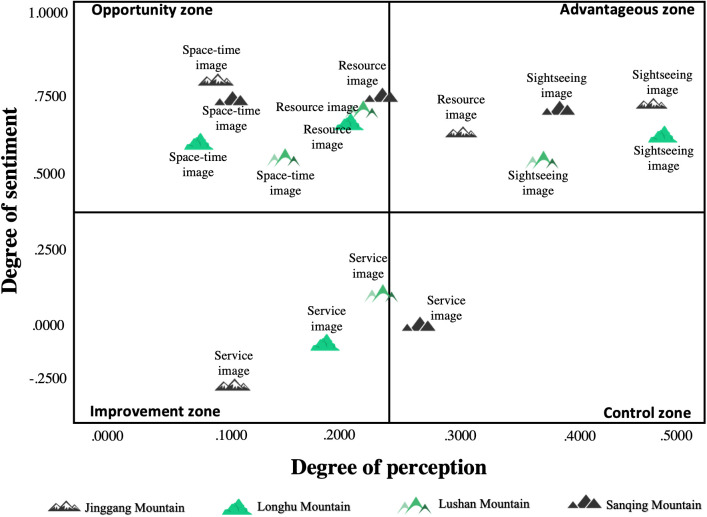
IPA analysis of PIs.

Based on the IPA analysis, the management of MSSs can focus on key priorities and identify the areas that require priority attention and improvement, thereby optimizing the efficiency of resource allocation. Moreover, this analysis is based on the needs and expectations of tourists, which significantly enhances their satisfaction. Fig 9 can be interpreted by considering the following aspects:

(1) The first quadrant is identified as an advantageous zone, representing prominent PIs and significant effectiveness of sentiment attachment. Sightseeing image are positioned in it for all MSSs, alongside the resources image of Jinggang mountain. This structure suggests that the sightseeing experience offered by MSSs is not only a central element of tourists’ PIs but also effectively evokes strong positive sentiments.
(2) The second quadrant represents an opportunity zone, representing a low level of perception but a high degree of sentiment engagement The resources image and space-time image of Sanqin Mountain, and Longhu Mountain, as well as the space-time image of Jinggang Mountain, all fall in this zone. Although these scenic spots have generated positive sentimental recognition among tourists, their perceived impact is relatively low. To improve this, strengthening the expressiveness, appeal, and overall influence of their image characteristics could be beneficial.
(3) The third quadrant is designated as an improvement zone, indicating areas where tourists exhibit low levels of both sentimental intensity and attention, thus requiring concentrated improvement. In particular, the service image across all MSSs need to be improved. Therefore, it should be a key focus on adding value to the service experience and optimizing service scenarios to increase operational and management standards.
(4) The fourth quadrant is defined as a control area, distinguished by high level of perception but a lower sentiment intensity. Sanqing mountain is uniquely located in this area. This positioning suggests a strong perception of the service image, but a corresponding lack of strong sentimental involvement of tourists. Thus, it is essential to explore new strategies for strengthening sentiment connections with tourists and to develop richer tourist products that provide greater sentiment value.

## 5. Discussion

### 5.1. Main findings

**Fig 10:**
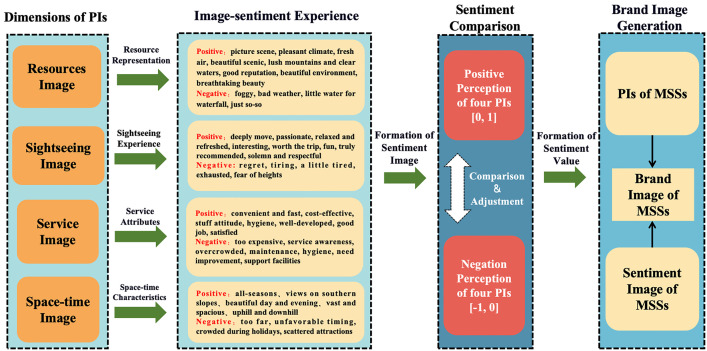
A generation model for the TBI of MSSs.

Taking four MSSs in Jiangxi Province as case studies, this research employs a comprehensive methodological approach that integrates perspectives from tourists’ PIs and sentiment analysis. Drawing upon a constructed model of TBI, the study illustrated the differentiation characteristics and formation mechanisms of TBI perception, offering new insights for the management and sustainable development of traditional MSSs. The main findings are as follows:

(1) The Tourist destination’s PIs function as a dynamic and multidimensional system, including four core concepts: resource image, sightseeing image, service image, and space-time image, alongside 14 related concepts. Unique functional characteristics represent each of these PIs. Additionally, its generation logic is manifested: the resources image serves as the foundation, the sightseeing image acts as the core carrier, the service image provides a guarantee, and the space-time image influences integrity. These four elements are dynamically adjusted through positive and negative feedback to form an overall PI.
(2) Regarding coarse-grained sentiment analysis, positive sentiments are significantly more than negative and neutral sentiments. Among them, Jinggang Mountain demonstrates the most positive sentiments. When analysing fine-grained sentiments, the space-time image is the most prominent, while negative sentiments are mainly concentrated in the service image. Moreover, there are significant differences in the degree of sentiment across different PIs and the various MSSs. For example, Sanqing Mountain exhibits the highest degree of sentiment in the resource image, while Lushan Mountain stands out in the service image. This provides empirical evidence for MSSs in differentiated marketing construction.
(3) Upon integrating the IPA analysis, significant differences among MSSs become apparent in both the degree of sentiment and perception. The majority of PIs are positioned in the advantageous and opportunity zones of the IPA matrix, with only the service image categorized into the improvement and control zones. By comparing the relative importance and priority of positive and negative sentiments as represented in the IPA figure, it is possible to understand the actual experiences of tourists under different PIs. This analysis offers robust empirical data and strategic guidance for enhancing the brand image of MSSs, and potentially for a broader range of scenic spots seeking to optimize their brand positioning.
(4) The aforementioned research indicates that the development of a tourist brand is fundamentally a process built upon the deep integration of PIs and associated sentiments, which is consistent with the findings of other research that destination PIs affect tourists’ satisfaction [74]. TBI formation occurs through the comparison and adjustment of positive and negative perceptions related to the various PIs. This formation process is crucial as it finally determines tourists’ cognitive and sentiment-based values concerning MSSs. Based on this logic, Fig 10 constructs a generation model for the MSSs’ TBI.

In addition, by comparing the perceived importance and priority of positive and negative sentiments associated with the four PIs, the model becomes possible to indicate the dynamic perception mechanism that tourists employ when forming an image of MSSs. This method can effectively and accurately reflect the trend of tourists’ psychological changes during the travel process. Specifically, this model quantifies the correlation between PI dimensions and sentiment degree, providing measurable indicators for objectively assessing MSSs’ brand image.

### 5.2. Theoretical contributions

The theoretical contributions of our research are mainly reflected in the following aspects, particularly in theoretical deepening, perspective expansion, and method innovation.

This research constructs a conceptual PIs model and a TBI generation model to enrich theoretical interpretations. Based on verifying destination brand equity theory [75], our research breaks through the limitations of static analysis and reveals the law of dynamic changes in perceptual images in tourist experience processes. This provides a theoretical framework for understanding the formation path of TBI in MSSs.

Meanwhile, this research verifies and deepens the applicability of the cognitive-affective theory. Compared with existing research, we analyzed differences in tourists’ cognition of different MSSs, confirmed that cultural and historical factors play a core role in shaping tourist satisfaction [76], and clarified the key impact on stimulating tourists’ positive sentiments. This is highly consistent with the view that cognition and sentiment jointly shape the destination image [77]. Additionally, through fine-grained sentiment analysis, we refine the explanation in existing research on “how cognition is specifically transformed into sentiment” and deepen the understanding of the interactive relationship between cognition and sentiment [78].

Furthermore, this research divides the PIs of MSSs into four core dimensions: resources, sightseeing, service, and space-time, and refines the system of PIs, providing a new perspective for the TBI theory (i.e., brand building should formulate differentiated strategies of “leveraging strengths and avoiding weaknesses”) and offering a new interpretation path for tourist loyalty research.

In addition, this research provides methodological support for tourism research in the era of big data. Our study used the LLM to process a large number of tourist comments and blogs. This method not only overcomes the limitations of traditional manual coding (such as low efficiency and intense subjectivity) but also provides replicable ideas for future tourism research based on existing studies [79,80].

### 5.3. Practical significance

For a long period, MSSs in Jiangxi province have contribute to a growing regional economy while remaining popular tourist destinations throughout China. To further promote the sustainable development capabilities of traditional MSSs, future construction efforts can prioritize the integration of PIs. This integration should focus on fully exploiting the strengths of these images, improving positive emotions among tourists, and creating new brand values. The aim is to develop a unique and innovative brand image for these destinations.

It is crucial to pay attention to the interactive effects of tourists’ PIs and to prioritize the creation of various brand connotations. In order to achieve this objective effectively, it is necessary to take a comprehensive consideration of tourists’ unique experiences with PIs, while building tourists’ trust and loyalty is crucial [81]. Furthermore, optimizing synergy among different PIs is essential. These combined efforts can facilitate a transition for tourist destinations, movie from “resource homogenization” to “brand connotation-oriented development,” and shifting from “resource competition” to “innovation competition.”

In addition, MSSs must adapt to local conditions by focusing on the most valuable PIs of tourist and re-evaluating their own brand positioning accordingly. Drawing from tourists’ PIs experiences, it becomes essential to optimize and innovatively develop new image identifiers specifically for traditional MSSs. Meanwhile, to effectively respond to the personalized requirements of diverse consumer groups, presenting distinctive, highly social, and interactive experiences is crucial, which can be achieved through multiple approaches. Efforts should be directed towards transforming basic tourism experiences—moving beyond simple “eating, lodging, transportation, and sightseeing”—and cultivating deeper perceptual experiences that consist of “savoring, enjoying, appreciating, and reflecting.” This transformation aims to highlight the lasting perceptual imprint of the tourist destination in the minds of visitors, thus enhancing both brand recognition and overall market competitiveness.

Furthermore, all MSSs should take advantage of their useful PIs to intentionally integrate sentimental elements into various aspects of tourist experience, including atmosphere design, cultural resource presentation. With the development tourism marketing, managers can use various of comprehensive methods [82,83]. By effectively stimulating tourists’ inner sentiments and carefully cultivating a human-centered atmosphere that genuinely resonates with their feelings, destinations can successfully cultivate positive sentiment experiences. These positive experiences play a crucial role in enhancing tourists’ sense of identity and cultivating loyalty to the destination. Meanwhile, it is necessary to proactively address the issues that lead to negative sentiments, even issues as seemingly ordinary as temperature [84], and drive the necessary transformation of MSSs, shifting them from being perceived as “simple product combinations” to “deeply integrated scenarios” that offers richer and more holistic experiences.

Moreover, the key is reflected by building distinctive PIs’ advantages and highlighting innovative brand values. To achieve this, tourism managers should, based on accurately identifying MSSs’ PIs and their sentiment distribution, leverage both “offline spaces” and “online spaces.” This utilization should be directed towards enhancing tourists’ overall tour experiences and effectively managing online social reputation. For example, Lushan Mountain has completed the “Cloud Tour” VR project by some companies (https://www.720yun.com/t/8bvkc98ypql?scene_id=60838700), Sanqing Mountain has completed the metaverse tourism system, and Flying Over 360° (https://www.wicherry.com/v/index.php?s=/addon/Diy/Diy/show/id/45/token/gh_fa5a854503ec.html). By effectively employing social media and AI technologies, these managers can propel MSSs to become new representatives and key drivers of high-quality development in the region, setting a benchmark for sustainable tourism and regional growth.

Finally, regarding the tourism cycle issue, especially the seasonality impact [85], efforts should be made to vigorously develop space-time resources, strengthen the construction of seasonal resources, and incorporate the surrounding areas into the scope to build a larger scenic space range, to enhance tourist perception of all-season and all-region tourism. Currently, Lushan Mountain has rime in winter and is a summer resort, successfully creating two well-known seasonal resource image landmarks. However, many other MSSs have not yet formed highly popular or attractive seasonal resource images. This is also consistent with the above research results, that is, no obvious seasonal words were found.

## 6. Research limitations

Despite efforts to ensure a comprehensive and complete data collection, we recognize that our study has limitation. In the current construction of MSSs, the PI related to seasonal differentiation is insufficient. This can be reflected in the tourists’ comments, which mentioned spatial and temporal perception aspects, but the seasonal indicators are still inadequate. This might be related to the insufficient development of all-time tourism in the current traditional MSSs construction. However, MSSs are indeed influenced by seasons. When the conditions for future research are mature, we will conduct studies on the relationship between seasons and the construction of MSSs. Moreover, the data samples were collected from online comments and blogs, providing plenty of research materials. However, these are only a part of all tourists, and there is a wider range of tourist groups to be studied. In future research, we can combine multimodal fusion data collection methods to address the issue of sample bias or conduct research on specific groups.
